# Alcohol‐mediated miR‐34a modulates hepatocyte growth and apoptosis

**DOI:** 10.1111/jcmm.13681

**Published:** 2018-06-05

**Authors:** Yoshifumi Iwagami, Jing Zou, Hongyu Zhang, Kevin Cao, Chengcheng Ji, Miran Kim, Chiung‐Kuei Huang

**Affiliations:** ^1^ Department of Medicine Rhode Island Hospital and The Warren Alpert Medical School of Brown University Providence Rhode Island USA

**Keywords:** Bcl2, CDK6, cyclin D1, Sirt1

## Abstract

MicroRNAs (miRs) have been recently shown to be heavily involved in the development of alcoholic liver disease (ALD) and suggested as a potential therapeutic target in ALD. The miR‐34a was consistently reported to be significantly elevated in several ALD rodent models, but it remains unclear how miR‐34a modulates the cellular behaviours of hepatocytes in ALD development and progression. This study aims to characterize alcohol‐induced miR‐34a impact on hepatocytes growth and apoptosis. The miRNA array was performed to assess changes in miRNA after chronic alcohol feeding. Liver and blood samples were used to examine ALD progression. The miR‐34a was overexpressed in human hepatocytes to evaluate its impact on cell growth and apoptosis. Real‐time quantitative PCR and Western blot were used to determine the growth and apoptosis molecular signalling pathways associated with miR‐34a. Alcohol feeding significantly promoted fatty liver progression, serum ALT levels, apoptosis and miR‐34a expression in rat liver. Overexpression of miR‐34a in human hepatocytes suppressed cell growth signallings, including c‐Met, cyclin D1 and cyclin‐dependent kinase 6 (CDK6). The miR‐34a might also inhibit the expression of sirtuin 1 (Sirt1) and its target, B‐cell lymphoma 2. Interestingly, the expression of miR‐34a reverses the suppressive effects of ethanol on cell growth. But, miR‐34a promotes hepatocyte senescence and apoptosis. Although the miR‐34a‐mediated down‐regulation of cell growth‐associated genes may contribute to cell growth retardation, other miR‐34a targets, such as Sirt1, may reverse this phenotype. Future studies will be needed to clarify the role of miR‐34a in ALD progression.

## 1 INTRODUCTION

Long‐term chronic alcohol abuse is associated with alcoholic liver disease (ALD). Depending on the histological features, ALD progression includes 3 stages: fatty liver, alcoholic hepatitis, and hepatic fibrosis or cirrhosis.^1^ Fatty liver often occurs without obvious symptoms and is reversible if patients stop chronic alcohol consumption. Alcoholic hepatitis is a severe form of ALD with an unknown mechanism of pathogenesis, because not every heavy drinker develops alcoholic hepatitis.^2^ ALD‐associated liver fibrosis/cirrhosis is the 12th leading cause of death in the United States.^3^ Although alcohol‐induced inflammatory responses and imbalanced microbiota have been suggested to participate in ALD development,^4^, ^5^, ^6^ the molecular pathogenesis of ALD remains unclear. Recently, non‐coding RNA, including miRNA, has emerged as a critical mediator in ALD progression.^7^ Investigating the effects of miRNA on cellular behaviours of hepatocytes may further clarify the molecular pathogenesis of ALD progression and development.

The miR‐34a is transcriptionally activated by p53 signalling^8^, ^9^ and has been demonstrated to promote cancer cell apoptosis, resulting in suppression of malignant tumour progression via transcriptional repression of oncogenes. The miR‐34a has been reported to suppress hepatocellular carcinoma invasion and migration by inhibiting c‐Met. The miR‐34a‐mediated down‐regulation of cyclin D1 and CDK6 has also been suggested to cause cell cycle arrest in lung carcinoma cells. In addition, miR‐34a, by suppressing MDM4/p53 signalling, might affect tumour malignant progression via positive feedback loop regulation.^10^, ^11^, ^12^ In terms of apoptosis, miR‐34a might inhibit sirtuin 1 (Sirt1) expression, which functions to regulate epigenetic gene silencing, to promote cell programme death.^13^ It also induces cellular apoptosis in neuroendocrine tumour cells by reducing the expression of B‐cell lymphoma 2.^14^ Thus, the systemic delivery of miR‐34a could induce apoptosis and abrogate growth of diffuse large B‐cell lymphoma.^15^ Furthermore, miR‐34a has been shown to be critically involved in B‐cell development by targeting Forkhead box transcription factor 1 (FoxP1).^16^ The following study further linked FoxP1 to nuclear factor kappa‐light‐chain‐enhancer of activated B‐cell‐associated apoptosis and suggested that miR‐34a‐mediated FoxP1 promotes B‐cell survival by directly repressing a set of 7 pro‐apoptotic genes.^17^ Intriguingly, recent studies revealed that miR‐34a is significantly up‐regulated in ALD rodent models as well as in non‐alcoholic fatty liver diseases,^18^, ^19^, ^20^ suggesting that miR‐34a may be a critical mediator in alcohol‐induced apoptosis.

As previous studies have demonstrated that miR‐34a is heavily involved in cell growth and apoptosis, investigating how miR‐34a modulates cell growth and apoptosis of hepatocytes may clarify the molecular pathogenesis of ALD development and progression. The current study aims to explore the direct impacts of miR‐34a on hepatocytes.

## 2 MATERIALS AND METHODS

### 2.1 Animal experiments

200 gram Long Evans (LE) male rats were purchased from Harlan Laboratories (South Easton, MA). ALD was induced in rat model following previous description.^21^ LE rats were fed with an isocaloric liquid diet for 7 days in the control group or the ethanol liquid diet, including 9% (calorie) ethanol for 2 days, 12.5% (calorie) ethanol for 2 days, and 25% (calorie) for 3 days in the ethanol group. LE rats were fed with a control diet in the control group or a 37% (calorie) ethanol diet in the control group for 8 weeks. Rats were weighed and sacrificed with CO_2_. Before the 8‐week treatment, experimental rats were fed one of the following diets; 7 days control isocaloric liquid diet or the ethanol liquid diet that ethanol‐derived calories was progressively increased from 9% (2 days) to 12.5% (2 days), and 25% (3 days). Rats were weighed and sacrificed with isoflurane. The liver and blood samples were harvested for haematoxylin and eosin (H&E) stain, TUNEL stain, AST assay, ALT assay and micro RNA assay. All animal experiments have been approved by IACUC animal care and use protocol committee of Lifespan Rhode Island Hospital.

### 2.2 Cells and reagents

Immortalized human hepatocytes were grown in DMEM Dulbecco's modified Eagle medium containing 10% foetal bovine serum, 1% L‐glutamine (Life Technologies, Gaithersburg, MD), 1% penicillin/streptomycin and 1% MEM non‐essential amino acids (Sigma Chemical Co, St Louis, MO). AST and ALT assay kits were purchased from Cayman Chemical. TUNEL staining kit (In Situ Cell Death Detection Kit) was purchased from Roche. The plasmids, pLL3.7_hsa‐miR‐34a (Plasmid #25791)^22^ and pLL3.7 (Plasmid #11795),^23^ were from Addgene. Micro RNA array chips were from Affymetrix Inc (Santa Clara, CA).

### 2.3 Senescence assay

The senescence assay kit (BioVision Inc.) was used to analyse human hepatocyte senescence. Basically, 10^5^ control‐treated or miR‐34a‐treated OUMS29 cells were seeded in 6‐well plate. Twenty‐four hours later, cells were fixed with fixation solution provided by the kit for 15 minutes and washed twice with PBS. After that, the senescence reaction mixture prepared following the instruction manual was added to the cultured cells and incubated overnight. The cells stained with blue colour were recognized as positive senescence cells.

### 2.4 MTS/MTT assay

The control‐treated or miR‐34a‐treated cells were plated in 96‐well plate at a concentration of 1000 cells/well. Twenty‐four hours later, cells were challenged with 0, 100 mmol/L ethanol or 100 mmol/L ethanol plus 20 ng/mL LPS for 1, 2 and 3 days. Medium containing those treatments were changed daily. The MTT solution prepared with 5 mg thiazolyl blue tetrazolium bromide (Sigma‐Aldrich) in PBS was added to the cells and incubated for 45‐60 minutes. The DMSO was added to the formed purple crystal and incubated with shaking for 30 minutes. The final dissolved purple solution was measured using an ELISA reader at 450 nm and 605 nm. The data were presented as OD450‐OD605.

### 2.5 Immunoblot analysis

Immunoblotting was performed as previously described.^24^ Basically, cells were lysed with RIPA buffers, and protein concentrations were quantified with BCA assay. The 50 μg of total protein was used to analyse gene changes in SDS gel. After proteins were transferred to the PVDF membrane, the target proteins were hybridized using specific antibodies, including CDK6 (sc‐177, 1:500), cyclin D1 (sc‐239, 1:250), Bcl2 (sc‐492, 1:500), GAPDH (sc‐25778, 1:3000), α‐tubulin (sc‐8035, 1:500) (all from Santa Cruz) and c‐Met (#4560, 1:1000, Cell Signaling). The specific 2nd antibodies either mouse (1:3000) or rabbit IgG (1:10 000) conjugated with horseradish peroxidase were used to visualize the target proteins.

### 2.6 Apoptosis induction

2 × 10^5^ control‐treated or miR‐34a treated OUMS29 cells were seeded in 6‐well plate. Twenty‐four hours later, cells were challenged with 0, 1, 2 μmol/L etoposide for 24 hours. Cells were collected with Trizol for mRNA analysis and fixed with methanol for TUNEL assay.

### 2.7 TUNEL staining

TUNEL staining was performed as previously described.^25^ Basically, the liver tissue slides were deparaffin with xylene and rehydrated with 100%, 95%, 75% ethanol and water. Tissue slides were then processed according to the instruction manual. After staining, the tissue samples were mounted with DAPI containing mounting medium and sealed with nail polish. The images were taken with a NIKON fluorescence microscope.

### 2.8 Caspase 3 activity assay

10^6^ human hepatocyte, OUMS29 cells transduced with control or miR‐34a containing lentivirus were seeded in 6‐well plate and challenged with 0, 100 mmol/L ethanol or 100 mmol/L ethanol plus 20 ng/mL lipopolysaccharide (LPS) for 48 hours. Cells were lysed with cell lysis buffer provided by the kit (Caspase‐3 Colorimetric Assay Kit, K106‐200, BioVision), incubated for 30 minutes and centrifuged at 16873g for 10 minutes. Protein supernatants were collected and quantified using the protein BCA assay. 50 μg proteins were used to perform caspase 3 activity, according to the instruction manual and the incubation time was overnight.

### 2.9 Real‐time quantitative PCR (RT‐qPCR)

RT‐qPCR was performed as previously described.^24^ The mRNA lysates were collected with Trizol reagent, and mRNA was prepared according to the instruction manual. The 1 μg mRNA was used to reversely transcript as cDNA. Specific primers listed in Table 1 and cDNA were used to perform an RT‐qPCR reaction using SYBR dye. The data were presented as relative mRNA expression.

**Table 1:** Primer sequences

Gene name	Primer sequence (5′→3′)
miR‐34a	Forward: CCAGCTGTGAGTGTTTCTTTG
	Reverse: CAGCACTTCTAGGGCAGTAT
c‐Met	Forward: AGCGTCAACAGAGGGACCT
	Reverse: TGAACCTCCGACTGTATGTCA
CCND1	Forward: GCTGCGAAGTGGAAACCATC
	Reverse: CCTCCTTCTGCACACATTTGAA
CDK6	Forward: TCTTCATTCACACCGAGTAGTGC
	Reverse: TGAGGTTAGAGCCATCTGGAAA
FoxP1	Forward: TGGCATCTCATAAACCATCAGC
	Reverse: GGTCCACTCATCTTCGTCTCAG
BIK	Forward: GACCTGGACCCTATGGAGGAC
	Reverse: CCTCAGTCTGGTCGTAGATGA
HRK	Forward: GGCAGGCGGAACTTGTAGGAAC
	Reverse: TCCAGGCGCTGTCTTTACTCTCC
TP63	Forward: AGCTGAGCCGTGAATTCAAC
	Reverse: CATACTGGGCATGGCTGTTC
TP53INP1	Forward: TTCCTCCAACCAAGAACCAGA
	Reverse: GCTCAGTAGGTGACTCTTCACT
Bcl2	Forward: GGTGGGGTCATGTGTGTGG
	Reverse: CGGTTCAGGTACTCAGTCATCC

### 2.10 H&E staining

Liver tissues were fixed in 10% neutralized formaldehyde for 24 hours and subjected to paraffin section at 5 μm thickness and subsequent H&E staining. Briefly, tissue slides were deparaffined and rehydrated as previously described.^26^ Processed slides were stained with haematoxylin for 2 minutes and differentiated under tap water for 5 minutes. Slides were incubated with 95% ethanol for 1 minute and stained with eosin for 30 seconds. After that, slides were incubated with 95% ethanol for 5 minutes 3 times, 100% ethanol for 3 minutes 2 times and 100% xylene for 5 minutes 3 times. Then, slides were mounted, using permount medium, with coverslip slides.

### 2.11 Micro RNA array

The miRNA was extracted using a miRNA isolation kit (Qiagen) from the livers of rats fed with either an isocaloric liquid control or an ethanol (37% of caloric) liquid diet for 8 weeks. Extracted miRNA was processed and hybridized with miRNA gene array chip (Affymetrix) following the instruction manual.

### 2.12 AST and ALT assays

Rat blood samples were collected via cardiac puncture and centrifuged for 30 minutes at 3000g. 50 μL of serum samples was used to perform AST and ALT assays following the instruction manuals.

### 2.13 Statistical analysis

Student *t* test was used to evaluate the statistical difference between control and treated groups. *P*‐value less than .05 was considered significant.

## 3 RESULTS

### 3.1 Chronic ethanol consumption causes liver injury and hepatic apoptosis

**Figure 1:**
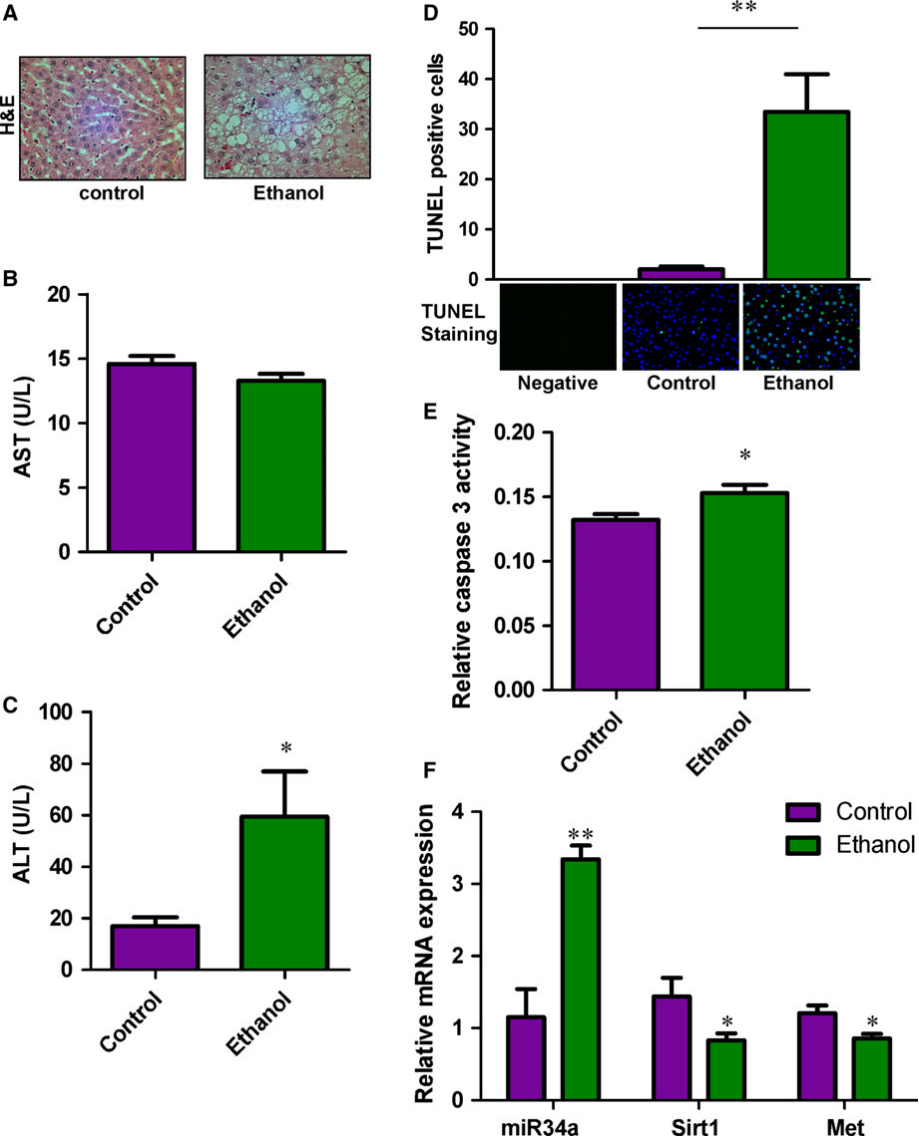
Chronic ethanol feeding induces steatosis and apoptosis in the rat liver. A, H&E staining of the rat livers. Liver injury and steatosis were observed in ethanol‐fed rats. B, AST C, and ASLT expression levels were determined in the serum samples harvested from control‐ and ethanol‐fed rats. n = 8. D, Apoptosis was measured in the livers of control‐ and ethanol‐fed rats using the TUNEL assay. Green fluorescence indicates the positive signal of apoptosis. DAPI stain, blue fluorescence, was used to show nuclear location. n = 5. E, The expression of miR‐34a, Sirt1 and Met was determined in the livers of control‐ and ethanol‐fed rats. n = 7. F, Relative caspase 3 activities were determined in control and ethanol‐fed rat liver samples. n = 5~7. *P*‐value was calculated from the student *t* test. *P < .05; **P < .01, when compared to control

Experimental rats were fed an isocaloric control or a 37% ethanol liquid diet for 8 weeks to mimic chronic alcohol consumption. As expected, chronic ethanol consumption induced hepatic injury and apoptosis in the experimental rats. The clear vacuole structure was observed in the ethanol‐fed rat livers but not in control‐fed ones (Figure 1A). Ethanol feeding also increased circulating ALT enzyme levels, TUNEL staining signals and caspase 3 activity in the rat livers. However, chronic ethanol feeding did not affect AST enzyme expression (Figure 1B‐E). These results, consistent with previous findings,^27^ suggest that the chronic ethanol feeding rat model does induce liver damage and apoptosis.

### 3.2 The miR‐34a is up‐regulated in the livers of ethanol‐fed rats

The correlation between miRNA and liver disease progression^28^ suggests certain miRNAs, including miR‐122, can be used as prognosis markers for ALD progression.^29^ The miRNA was extracted from the livers of control‐fed and ethanol‐fed rats and evaluated for miRNA profile differences using a miRNA array. As shown in Table 2, there was around fourfold increase in miR‐34a expression of ethanol‐fed rats. The array data of miR‐34a were further validated with qRT‐PCR (Figure 1F). Additionally, the reported miR‐34a targets, Sirt1 and c‐Met, were demonstrated down‐regulated in ethanol‐fed rat livers. As the change in miR‐34a was relatively large when compared to other altered miRNAs and miR‐34a has been reproducibly demonstrated up‐regulated among other varieties of liver diseases,^18^, ^20^, ^30^ it is critical to determine the impact of miR‐34a in ALD progression.

**Table 2:** miRNA profiles of control versus ethanol‐fed rat livers

Name	Control	Ethanol	*P*‐value
miR‐34a	1.08	3.82	.001
miR‐455	1.00	2.04	.047
miR‐182	1.01	1.92	.003
miR‐351	1.01	1.76	.037
miR‐423	1.02	1.60	.039
miR‐23b	1.01	1.58	.008
miR‐532‐3p	1.00	1.56	.029
miR‐200a	1.01	1.55	.018
miR‐935	1.00	1.48	.032
miR‐3563	1.00	1.45	.004
miR‐203	1.00	0.80	.012
miR‐3085	1.00	0.80	.032
miR‐3575	1.00	0.78	.030
miR‐211	1.00	0.78	.002
miR‐376c	1.00	0.77	.008
miR‐541	1.00	0.76	.041
miR‐547	1.00	0.75	.015
miR‐130a	1.01	0.67	.024
miR‐708	1.00	0.61	.043
miR‐3559‐3p	1.01	0.54	.034

### 3.3 miR‐34a inhibits cell growth signalling pathways in human hepatocytes

**Figure 2:**
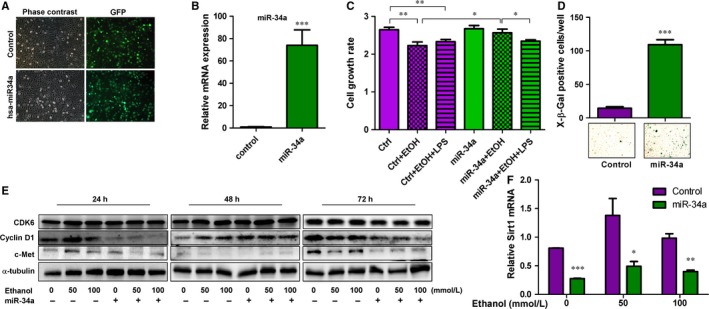
Overexpression of miR‐34a in human hepatocytes influences cell growth and senescence. A, Images of phase contrast and green fluorescence protein (GFP) were shown in human hepatocytes manipulated with control and pLL3.7‐has‐miR‐34a plasmids via lentiviral system which contains GFP. B, Expression levels of miR‐34a were determined in treated human hepatocytes, n = 3. C, Relative cell growth rates of human hepatocytes treated as indicated for 3 days were measured, n = 6. D, Senescence was detected, using X‐β‐gal staining, in human hepatocytes transduced with control lentivirus or miR‐34a containing lentivirus. Blue colours indicate senescent cells, n = 3. E, Protein expression levels of CDK6, cyclin D1, c‐Met and α‐tubulin were determined in control‐treated or miR‐34a‐treated hepatocytes in the presence of 0, 50, 100 mmol/L ethanol for 24, 48 and 72 h. F, The Sirt1 mRNA expression levels were measured in human hepatocytes treated as indicated for 72 h, n = 4. *P*‐value was calculated using student *t* test. *P <* *.05; **P <* *.01; ***P <* *.001, when compared to control

To determine how miR‐34a affects liver functions in ALD, the miR‐34a was expressed in human hepatocytes via the lentiviral system containing green fluorescence protein (GFP). The successful delivery of miR‐34a was verified with GFP with more than 90% of cells positive for GFP signals (Figure 2A). Additionally, overexpression of miR‐34a was increased about 80‐fold as verified using RT‐qPCR (Figure 2B). We then used MTS/MTT assay to analyse how miR‐34a affects hepatocyte growth. Intriguingly, 100 mmol/L ethanol and 100 mmol/L ethanol plus 20 ng/mL LPS challenge could significantly inhibit human hepatocyte growth, but the expression of miR‐34a would reverse the ethanol's effects on hepatocyte in the absence of 20 ng/mL LPS. These interesting results are actually consistent with the previous study that expression of pre‐miR‐34a promotes cell survival and anchorage‐independent cell, whereas anti‐miR‐34a inhibits cell proliferation.^18^ As miR‐34a‐mediated senescence has been recently found to participate in disease progression of ALD and cancers,^30^, ^31^ we also determined whether miR‐34a promotes hepatocyte senescence. The results suggest that expression of miR‐34a substantially increases senescent cells (Figure 2D). To further clarify how miR‐34a affects cell growth and senescence, we then evaluated whether those miR‐34a targets associated with cell growth, including c‐Met, cyclin D1 and CDK6, are decreased upon miR‐34a treatment.^10^, ^12^ We focus on c‐Met, cyclin D1 and CDK6, because they have been reported to be crucial mediators in liver disease development and progression.^32^, ^33^, ^34^ Consistent with the previous findings,^10^, ^12^ we found that miR‐34a significantly suppressed c‐Met and cyclin D1 expression in human hepatocytes challenged with 50 and 100 mmol/L ethanol for 24 and 72 hours. Additionally, miR‐34a slightly inhibited CDK6 expression in hepatocytes treated with 0, 50, 100 mmol/L ethanol for 72 hours (Figure 2E). But, these results contradict to our finding that miR‐34a could reverse the suppressive effects of ethanol on hepatocyte growth. To understand how miR‐34a rescues the impacts of ethanol on hepatocyte growth, we have chosen to analyse Sirt1 expression in control‐treated and miR‐34a‐treated hepatocytes challenged with 0, 50, 100 mmol/L ethanol for 72 hours, as Sirt1 has been implicated to be involved in promoting cell survival and cell growth in hepatocytes.^18^ Our results show that expression of miR‐34a significantly reduced Sirt1 expression. Thus, Sirt1 may be one of the potential reasons causing that miR‐34a reverses the suppressive effects of ethanol on hepatocyte growth.

### 3.4 FoxP1‐mediated apoptotic signalling pathways are not altered by miR‐34a

**Figure 3:**
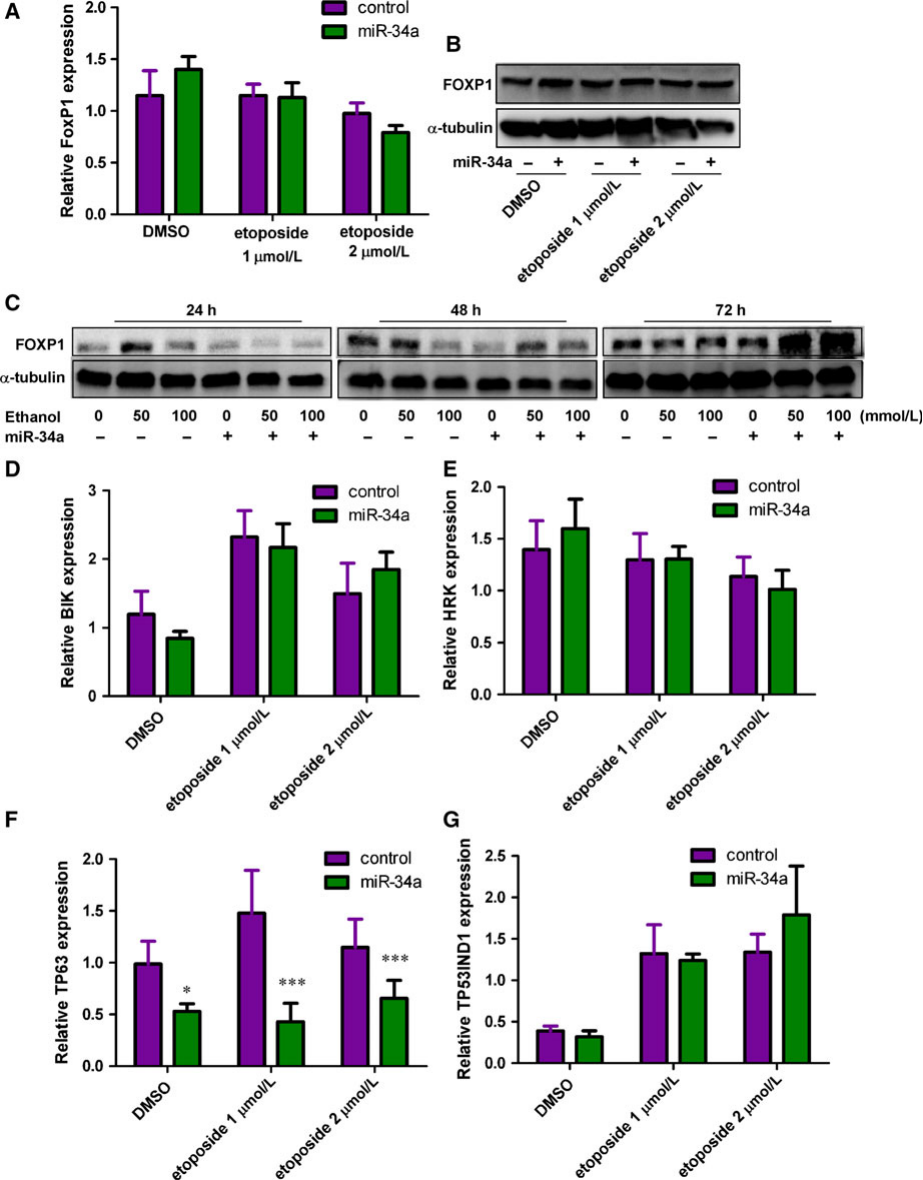
The impact of miR‐34a on FoxP1‐mediated apoptotic signalling pathways. A, FoxP1 mRNA expression levels were measured in control‐ or miR‐34a‐treated human hepatocytes challenged with DMSO, 1 and 2 μmol/L etoposide for 24 h. n = 6. B, Immunoblotting results of FoxP1 and α‐tubulin were determined in human hepatocytes treated as indicated for 24 h. C, FOXP1 and α‐tubulin were determined in control‐ and miR‐34a‐treated human hepatocytes challenged with 0, 50, 100 mmol/L ethanol for 24, 48 and 72 h. The mRNA expression levels of FoxP1‐mediated downstream apoptotic signalling molecules, including the BH3 only proteins D, BIK and E, Harakiri (HRK) as well as the p53‐regulatory proteins F, tumour protein P63 (TP63) and G, tumour protein P53 inducible nuclear protein 1, were determined in human hepatocytes treated as indicated. (TP53INP1). n = 6. *P*‐value was calculated using student *t* test. *P <* *.05; ***P <* *.001, when compared to control

FoxP1, a critical mediator involved in B‐cell development,^16^ is one of the validated miR‐34a target genes. It has been recently found to participate in promoting survival of B cell through inhibiting pro‐apoptotic molecules.^17^ As such, ethanol‐induced miR‐34a might inhibit FoxP1 expression to result in apoptosis of hepatocytes. FoxP1 and its downstream molecules, including BIK, HRK, TP63 and TP53INP1, were measured to evaluate the effects of miR‐34a on FoxP1‐mediated apoptotic signalling pathways. Unexpectedly, overexpression of miR‐34a did not have significant impacts on the expression levels of FoxP1 (Figure 3A,B) in human hepatocytes challenged with 0, 1, 2 μmol/L etoposide. Although expression of miR‐34a slightly inhibited FOXP1 expression in human hepatocytes challenged with 50 and 100 mmol/L ethanol for 24 hours, these effects were lost upon prolonged challenges (Figure 3C). The FOXP1 downstream targets, including BIK, HRK and TP53IND1, were also found not affected by miR‐34a (Figure 3D,E, and G). Although TP63 was significantly reduced by miR‐34a (Figure 3F), this may be a direct effect of miR‐34a rather than through FoxP1 as FoxP1 was not altered by miR‐34a treatment.

### 3.5 Overexpression of miR‐34a inhibits the expression of anti‐apoptotic protein, Bcl2

**Figure 4:**
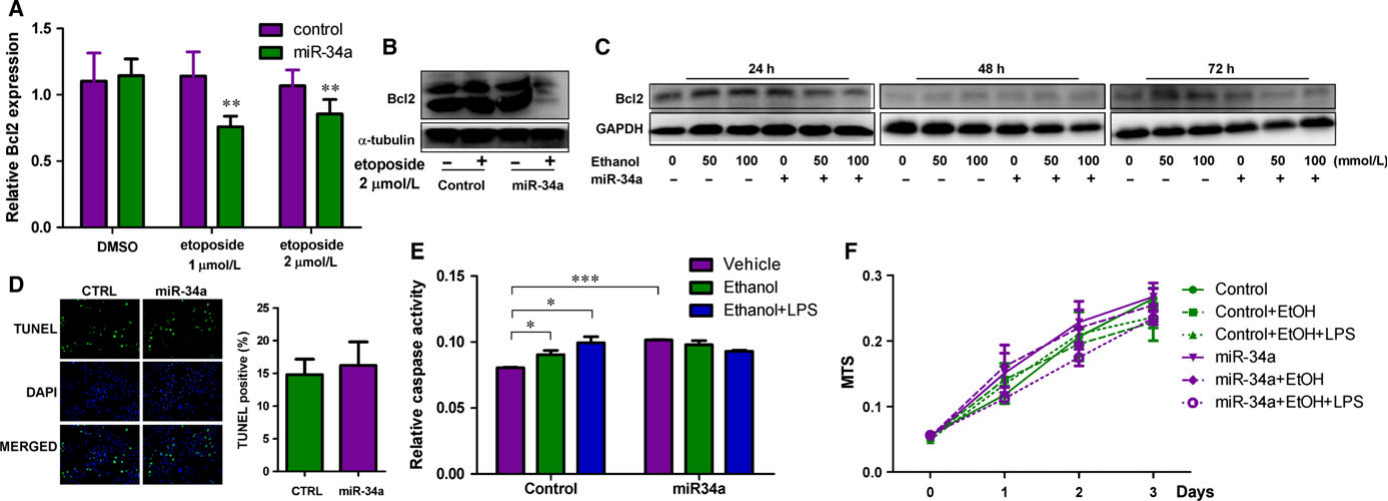
Overexpression of miR‐34a affects human hepatocyte apoptosis. A, The mRNA and B, protein expression levels of Bcl2 were determined in control and miR‐34a human hepatocytes treated with DMSO, 1 and 2 μmol/L etoposide for 24 h. α‐tubulin served as loading control. C, Bcl2 and GAPDH were determined in human hepatocytes treated as indicated. D, Apoptotic signals of control and miR‐34a human hepatocytes treated with DMSO, 1 and 2 μmol/L etoposide were evaluated using the TUNEL assay. Green fluorescence indicates the positive signal of apoptosis. DAPI signal shows nuclear location. The right panel is the quantitative graph of apoptosis. n = 4. E, Caspase 3 activities were determined in control‐ and miR‐34a‐treated human hepatocytes challenged with vehicle control (0 mmol/L ethanol), 100 mmol/L ethanol and 100 mmol/L ethanol plus 20 ng/mL lipopolysaccharides (LPS) for 48 h. F, The relative cell growth was measured, using the MTS assay, in control‐ and miR‐34a‐treated human hepatocytes challenged with 0, 100 mmol/L ethanol and 100 mmol/L ethanol plus 20 ng/mL LPS for 1, 2 and 3 days, n = 6. *P*‐value was calculated using student *t* test. *P < .05; **P < .01; ***P < .001; when compared with the control.

Bcl2 was reported to be another apoptotic signal that might be regulated by miR‐34a.^35^ The mRNA expression levels of Bcl2 were examined in human hepatocytes treated with control and miR‐34a. As shown in Figure 4A, Bcl2 mRNA expression levels were unchanged upon miR‐34a expression in the control condition. However, miR‐34a did inhibit Bcl2 mRNA expression when hepatocytes were treated with etoposide to induce apoptosis. Consistent with mRNA expression, miR‐34a suppressed Bcl2 protein expression in the presence of AI for 24 hours (Figure 4B). These phenotypes were reproducible when hepatocytes were challenged with 50 and 100 mmol/L ethanol for 24 and 72 hours (Figure 4C). To further determine whether miR‐34a alters apoptosis of hepatocytes, the TUNEL assay was used to examine DNA fragmentation, an apoptotic phenotype, in human control‐treated and miR‐34a‐treated hepatocytes challenged with vehicle or etoposide for 24 hours. Interestingly, miR‐34a, unlike its promoting role in cancer apoptosis,^8^, ^9^ did not alter apoptosis either in the presence or absence of etoposide (Figure 4D and non‐detectable). We then used another approach, caspase 3 activity, to evaluate the impact of miR‐34a on apoptosis. As shown in Figure 4E, treatments with 100 mmol/L ethanol or 100 mmol/L ethanol plus 20 ng/mL LPS for 48 hours could enhance caspase 3 activities. Expression of miR‐34a could increase caspase 3 activity as well. However, miR‐34a could not further promote caspase 3 activity in the presence of 100 mmol/L ethanol or 100 mmol/L ethanol plus 20 ng/mL LPS. Finally, we evaluated whether miR‐34a affects cell growth using MTS/MTT assay and found that miR‐34a consistently inhibited cell growth in the presence of 100 mmol/L ethanol plus 20 ng/mL LPS at days 2 and 3. But, miR‐34a could reverse the ethanol's suppressive effects on hepatocyte at days 2 and 3. These results suggest that miR‐34a may differentially modulate hepatocyte apoptosis under different types of stress conditions (Figure 4F).

## 4 DISCUSSION

The miR‐34a has been thought to be a tumour suppressor in several cancers due its direct transcriptional target of p53, a well‐documented tumour suppressor.^8^, ^9^ In earlier studies, miR‐34a was reported to be directly regulated by p53 activity through transcriptional regulation. This p53‐mediated miR‐34a expression was demonstrated to be involved in cell apoptosis of cancer cells.^13^ In addition, several predicted miR‐34a targets, including cyclin D1, CDK6 and c‐Met 10, 12 which participate in cell growth control, have been confirmed to be transcriptionally regulated by miR‐34a and their down‐regulations through miR‐34a did suppress cancer cell progression. To confirm the in vivo function of miR‐34a in malignant cancer progression, the genetic knockout of miR‐34a mouse model was generated. The study suggested that miR‐34a knockout might suppress lung cancer progression through a positive feedback effect on p53 signalling pathway by inhibiting HDM4, a negative regulator of p53 signalling.^11^ However, another study using triple miR‐34 knockout mice showed that deletion of miR‐34a, b and c has no significant impacts on p53‐mediated responses, including malignant cancer progression.^36^ Although the role of miR‐34a in cancer malignant progression might be controversial, there is one study investigating miR‐34a in cardiac fibrosis and demonstrating that knockout of miR‐34a might reduce fibrosis progression through the protein, serine/threonine‐protein phosphatase 1 regulatory subunit 10.^37^ As fibrosis is a later stage of alcoholic liver diseases, the results might link miR‐34a to liver fibrosis as well.

The miR‐34a has been identified to be up‐regulated in different kinds of liver diseases, including non‐alcoholic steatohepatitis (NASH) and ALD but down‐regulated in hepatocellular carcinoma.^18^, ^20^, ^38^ Previous studies have linked p53 to miR‐34a in high‐fat diet‐induced NASH model as well as in human patients with non‐alcoholic liver diseases.^20^, ^39^ The miR‐34a‐mediated liver disease progression was through controlling protein acetylation via inhibiting the expression of Sirt1, also known as NAD‐dependent deacetylase sirtuin‐1. Elevation of miR‐34 leads to down‐regulation of SIRT1, which results in acetylation of PGC1a and dephosphorylation of AMPK, therefore alters metabolism. In addition, miR‐34a‐mediated Sirt1 has been previously demonstrated to participate in cancer apoptosis,^13^ suggesting that miR‐34a‐mediated apoptosis may be one of the reasons that cause hepatocyte apoptosis.

As accumulating evidence suggests that miR‐34a may induce hepatocyte apoptosis and exacerbate ALD progression, we therefore evaluated whether the expression of miR‐34a may directly affect human hepatocyte expression. Although our data showed that cyclin D1, c‐Met, CDK6 and Bcl2 were down‐regulated upon miR‐34a treatment, cell growth and apoptosis of human hepatocytes were differentially altered. These unexpected findings complicated the hypothesized role of miR‐34a in ALD progression. Several different kinds of cells have been suggested to be involved in ALD progression. Among them, inflammatory cells and hepatic stellate cells are extensively investigated due to their influences in alcohol‐associated hepatitis and liver fibrosis. The miR‐34a and miR‐34c were found to promote hepatic stellate cell activation, which is one of the critical processes involved in liver fibrosis. The miR‐34a and miR‐34c could transcriptionally repress peroxisome proliferator‐activated receptor γ expression to promote hepatic stellate cell activation.^40^ However, miR‐34a could suppress LPS‐induced inflammatory responses in murine macrophages, which has been demonstrated to be a critical mediator in liver fibrosis.^4^ Despite these controversial in vitro findings could not explain how miR‐34a modulates ALD progression, there has been a report demonstrating that suppression of miR‐34a in high‐fat diet‐fed mice significantly reduces fatty liver progression.^41^ The underlying mechanisms of NASH and ALD may be due to different causes. However, miR‐34a expression has been shown up‐regulated in both NASH and ALD in vivo, suggesting that miR‐34a may be the potential therapeutic target for ALD. Indeed, a recent study has demonstrated that treating ALD mouse model with pre‐miR‐34a could alleviate ALD progression.^30^

Non‐coding RNAs including microRNAs have been suggested to be prognosis markers and even to be the therapeutic approach in malignant cancer progression.^42^ Although miR‐34a has been shown to be elevated in NASH and ALD and be a therapeutic target in fatty liver disease and ALD, it should be aware that miR‐34a is a tumour suppressor in malignant cancer progression. Further investigations will be needed in order to determine the precise time‐points of treatment with miR‐34a antagonist and whether it is worth it to target miR‐34a in NASH and ALD.

## CONFLICT OF INTEREST

The authors who have taken part in this study declared that they do not have anything to disclose regarding funding or conflict of interest with respect to this manuscript.
